# Caspases from scleractinian coral show unique regulatory features

**DOI:** 10.1074/jbc.RA120.014345

**Published:** 2020-08-11

**Authors:** Suman Shrestha, Jessica Tung, Robert D. Grinshpon, Paul Swartz, Paul T. Hamilton, Bradford Dimos, Laura Mydlarz, A. Clay Clark

**Affiliations:** 1 Department of Biology, University of Texas at Arlington, Arlington, Texas, USA; 2 Department of Molecular and Structural Biochemistry, North Carolina State University, Raleigh, North Carolina, USA; 3 Department of Plant and Microbial Biology, North Carolina State University, Raleigh, North Carolina, USA

**Keywords:** caspase, cysteine protease, allosteric regulation, substrate specificity, coral apoptosis, coral immunity, functional divergence, substrate selection, CARD-caspase, apoptosis

## Abstract

Coral reefs are experiencing precipitous declines around the globe with coral diseases and temperature-induced bleaching being primary drivers of these declines. Regulation of apoptotic cell death is an important component in the coral stress response. Although cnidaria are known to contain complex apoptotic signaling pathways, similar to those in vertebrates, the mechanisms leading to cell death are largely unexplored. We identified and characterized two caspases each from *Orbicella faveolata*, a disease-sensitive reef-building coral, and *Porites astreoides*, a disease-resistant reef-building coral. The caspases are predicted homologs of the human executioner caspases-3 and -7, but OfCasp3a (*Orbicella faveolata* caspase-3a) and PaCasp7a (*Porites astreoides* caspase-7a), which we show to be D*XX*Dases, contain an N-terminal caspase activation/recruitment domain (CARD) similar to human initiator/inflammatory caspases. OfCasp3b (*Orbicella faveolata* caspase-3b) and PaCasp3 (*Porites astreoides* caspase-3), which we show to be V*XX*Dases, have short pro-domains, like human executioner caspases. Our biochemical analyses suggest a mechanism in coral which differs from that of humans, where the CARD-containing D*XX*Dase is activated on death platforms but the protease does not directly activate the V*XX*Dase. The first X-ray crystal structure of a coral caspase, of PaCasp7a determined at 1.57 Å resolution, reveals a conserved fold and an N-terminal peptide bound near the active site that may serve as a regulatory exosite. The binding pocket has been observed in initiator caspases of other species. These results suggest mechanisms for the evolution of substrate selection while maintaining common activation mechanisms of CARD-mediated dimerization.

Apoptotic cell death is thought to be a unique characteristic of metazoans, although its evolutionary origins are unclear. Although caspases from human cells and model organisms such as *Caenorhabditis elegans* and *Drosophila* have been well-studied both biochemically and structurally (1–6), little is known about caspase activity and regulation in other basal species (7). *C. elegans* (3, 6) and *Drosophila* (8) were some of the first invertebrate caspases to be characterized, but they have proved to be poor models for studying the evolution of the vertebrate apoptotic network as their networks utilize fewer caspases and regulatory proteins compared with higher eukaryotes. *C. elegans*, for example, utilizes only one effector caspase (CED-3), which also bears a CARD-motif necessary for its activation (9). Moreover, cytochrome *c* is not involved in the formation of the apoptosome in *Drosophila*, indicating that this organism lacks the intrinsic pathway found in humans (2). In contrast, it now appears that vertebrates have retained many characteristics of the apoptotic machinery found in sponges, sea anemone, and coral (10–12). Genomic studies of cnidarians, the sister group to the bilateria, revealed many genes that were previously thought to have been vertebrate innovations, demonstrating that the extensive gene loss in *C. elegans* and in *Drosophila* resulted in apoptotic pathways that do not reflect the characteristics of ancestral metazoans (13, 14). Basal metazoans, which appear to have a full complement of apoptotic signaling molecules, may therefore be more relevant to the evolutionary pathways of vertebrate apoptotic networks.

Cnidarians including reef-building corals from the genus Scleractinia are ecologically important organisms that are on the decline (15) and cell death has been indicated to be important in these processes (16). The two primary drivers of coral declines are marine diseases affecting reef-building corals (17) as well as temperature-induced loss of the coral's symbiont known as bleaching (15). Coral possess an innate immune system that both defends the animals against pathogenic organisms and also serves as general stress responses (18). Therefore, the coral immune system is critical in the response of these organisms to both coral diseases and bleaching. Activation of the innate immune system activates apoptotic pathways (19); however, to date very few functional studies have been performed to characterize caspase structure and subsequent function in corals (20).

There are several examples pointing to the importance of apoptotic pathways and caspases in coral survival to both disease and temperature stress. An increase in expression of apoptosis-related genes was detected in a diseased Caribbean soft coral resulting in a visible inflammatory response (black-melanized appearance) (21). Also, executioner caspase genes were up-regulated in the branching coral Acropora infected with white band disease (22). Several studies have gleaned important insights into coral apoptosis post-temperature stress by demonstrating that corals activate cell death responses following expulsion of their algal symbiont (19, 23–25). Specifically, the anti-apoptotic protein Bcl-2 in *Acropora millepora* is up-regulated during temperature stress (26), indicating that this species likely has an intrinsic apoptosis mechanism as well as mechanisms to regulate this process. Interestingly, it was shown that application of caspase inhibitors can prevent the death of bleached coral (10). Collectively, the data show the potential for complex apoptotic signaling pathways in coral but data on activation and control mechanisms, and how they compare with those in vertebrates, are lacking because of a dearth of biochemical characterization.

To gain insight into caspase activity and regulation in coral, we expressed and characterized two caspases each from two species of Caribbean reef-building corals, *Orbicella faveolata* and *Porites astreoides*. The two coral species are found on opposite ends of the stress-tolerance spectrum where the disease-susceptible *O. faveolata* activates caspase-mediated apoptotic pathways upon immune challenge, whereas the disease-tolerant *P. astreoides* activates an adaptive autophagic response (19). These findings indicate that understanding the apoptotic machinery in corals likely has significant implication in understanding species stress tolerance. In this investigation we describe the structural composition of each species' caspase repertoire, and we use these data to functionally characterize both initiator and effector caspases from both species. Two proteins referred to as PaCasp7a and OfCasp3a based on sequences similarity to human caspases contain CARD motifs at the N terminus, an unusual combination that has not been observed in caspases-3 or -7 enzymes from higher eukaryotes, and indeed these proteins function as initiator caspases. Additionally, two proteins PaCasp3 and OfCasp3b show canonical caspase-3/7 structural organization, with short pro-domains, and possess effector caspase function. We describe the first biochemical characterization of coral caspases and show that the PaCasp3 and OfCasp3b enzymes are not activated directly by the CARD-containing PaCasp7a and OfCasp3a, respectively. We also report the first X-ray crystal structure of a coral caspase, that of PaCasp7a determined at 1.57 Å resolution, which reveals an N-terminal peptide bound near the active site that may serve as a regulatory exosite. Overall, we find support for complex apoptotic mechanisms in these early metazoans, where the cellular machinery for both intrinsic and extrinsic apoptosis has ancient evolutionary origins.

## Results

### Caspases in two coral species: Phylogenetic analysis and domain organization

**Figure 1.**
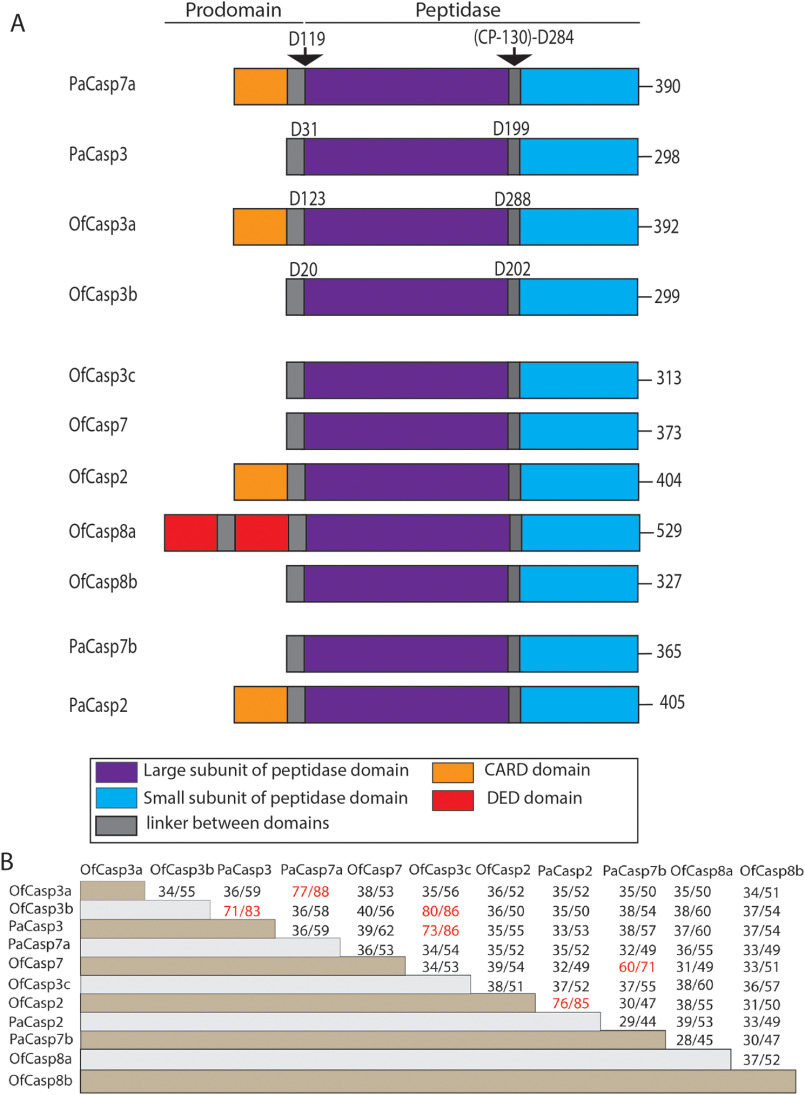
**Domain organization and sequence comparison among caspases of *O. faveolata* and *P. astreoides*.** *A*, domain organization of caspases in *O. faveolata* and *P. astreoides*. Processing site between large and small subunit, and after pro-domains are noted in biochemically characterized caspase. *B*, protein sequence identity (%) and similarity (%) among coral caspases.

We examined seven caspase genes from *O. faveolata* based on sequences obtained from previous transcriptomic and genomic data (Fig. S1 and Table S1) (19). The caspases were named based on the E-value from BLAST as well as the sequence similarity to the human orthologs. Results from examining the sequence homology and domain organization suggest that three of the caspases are apoptotic initiators and four are apoptotic effectors in *O. faveolata* (Fig. 1*A*). The sequence identities of the seven caspases compared with most human caspases are low, only ∼35% (Table 1), so it is difficult to determine the nature of each coral caspase based solely on sequence comparisons with human orthologs. In addition, two caspases from *O. faveolata* contain an N-terminal caspase activation and recruitment domain (CARD) motif, similar to those in HsCasp2 and HsCasp9, and one caspase contains tandem death effector domain (DED) motifs, similar to that found in HsCasp8 (Fig. 1*A*). The remaining four proteins show domain organization similar to the human effector caspases, with short pro-domains (Fig. 1*A*).

**Table 1:** **Protein sequence identity/similarity (%) with human caspases**

	HsCasp3	HsCasp7	HsCasp6	HsCasp2	HsCasp8	HsCasp10	HsCasp9
OfCasp7	37/54	38/52	32/49	28/43	34/50	33/53	34/50
OfCasp3c	36/58	35/56	35/52	32/48	37/53	37/54	33/49
OfCasp3b	35/60	32/57	33/52	32/49	37/55	33/53	34/50
OfCasp3a	47/69	45/65	38/54	29/48	39/54	39/55	28/46
OfCasp2	37/53	41/55	35/46	33/52	34/52	35/52	32/48
OfCasp8a	39/56	39/53	34/48	35/53	32/58	30/49	34/51
OfCasp8b	33/56	31/50	31/49	32/52	35/51	34/52	32/46
PaCasp3	36/58	37/59	36/56	33/49	37/54	34/54	35/50
PaCasp7a	43/65	44/60	36/53	28/46	37/53	37/53	28/44
PaCasp7b	38/54	37/52	34/49	29/46	31/48	30/50	33/48
PaCasp2	39/53	38/52	33/48	33/50	35/53	35/52	32/49

In the case of *P. astreoides*, four caspase sequences consisted of two initiator-like caspases (called PaCasp7a and PaCasp2) and two effector-like caspases (called PaCasp7b and PaCasp3) (Fig. 1*A* and Fig. S1). Similar to the results for *O. faveolata*, the caspase sequences from *P. astreoides* also have only ∼35% identity with human caspases, regardless of comparisons to initiator or effector caspases (Table 1). The sequences from the two coral species displayed much higher identity to putative homologs in the other coral species. For example, PaCasp7a has a 77% sequence identity with OfCasp3a, whereas PaCasp3 has 71 and 73% sequence identity, respectively, with OfCasp3b and OfCasp3c. Likewise, PaCasp2 demonstrates 76% sequence identity with OfCasp2, and PaCasp7b shares 60% identity with OfCasp7 (Fig. 1*B*).

**Figure 2.**
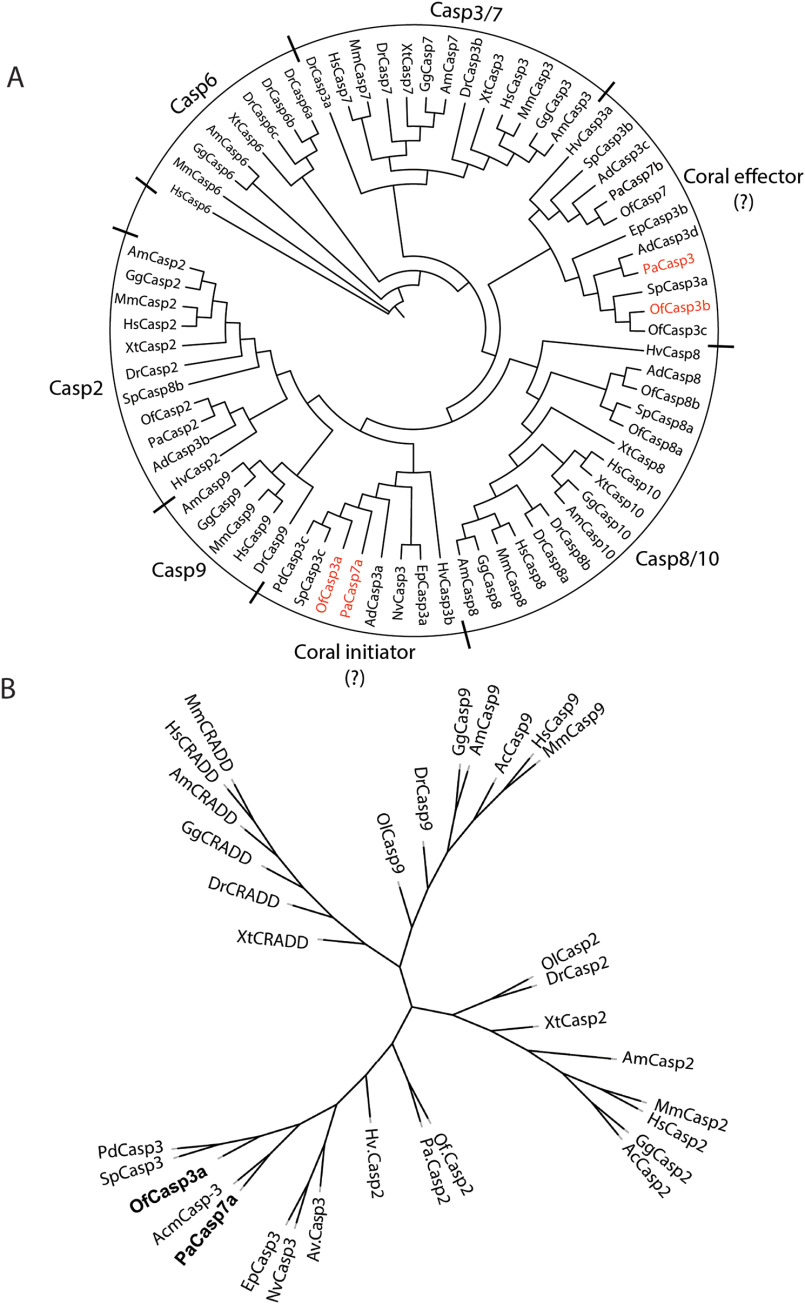
**Coral caspase phylogenetic analysis.** *A*, phylogenetic tree of cnidarian and vertebrate caspases: *Orbicella faveolata* (*Of*), *Porites astreoides* (*Pa*), *Pocillophora damicornis* (*Pd*), *Stylophora pistillata* (*Sp*), *Nematostella vectensis* (*Nv*), *Exaiptasia pallida* (*Ep*), *Hydra vulgaris* (*Hv*), *Acropora digitophora* (*Ad*), *Homo sapiens* (*Hs*), *Mus musculus* (*Mm*), *Gallus gallus* (*Gg*), *Alligator mississippiensis* (*Am*), *Xenopus laevis* (*Xl*), *Danio rerio* (*Dr*). Accession number of all used sequences are shown in Tables S1 and S2. *B*, phylogenetic analysis of CARD domains of caspases and CRADDs between cnidarians and vertebrates.

A phylogenetic analysis of cnidarian and vertebrate caspases demonstrated that cnidarian caspases cluster in separate groups (Fig. 2*A*). All of the short pro-domain caspases, including PaCasp3 and OfCasp3b, cluster together between vertebrate effector (caspases-3/7) and initiator (caspases-8/10) caspases. Interestingly, the comparative genomics and phylogenetic analyses suggest that short cnidarian caspases, that is, those lacking a CARD or DED, share a common ancestor with vertebrate effector caspases-3 and -7 and with initiator caspases-8 and -10 (Fig. 2*A*). Homologs of caspase-8 in coral share the same clade with vertebrate caspases-8 and -10 and the CARD-containing OfCasp2 and PaCasp2 clustered with vertebrate caspase-2. With the exceptions of OfCasp2 and PaCasp2, the other CARD-containing coral caspases cluster with OfCasp3a and PaCasp7a and segregate into a different clade, although they share a common ancestor with vertebrate caspases-2 and -9.

We analyzed the CARD motifs of cnidarian caspases independently of the protease domains and compared them to the CARD motifs of vertebrate caspases-2 and -9 as well as that of caspase-2 and RIPK1 domain containing adaptor with death domain (CRADD) motifs, which recruit caspase-2 to the PIDDosome (27) (Fig. 2*B*). The CARD motifs of coral caspases-3 and -7 cluster together but are more closely related to the CARD of caspase-2 than those of caspase-9 or CRADD. Based on this analysis, there appear to be many CARD-containing caspase-3–like proteins in cnidaria. At present, it is not clear why CARD-containing caspase-3–like proteins provide an advantage for coral development and/or symbiosis because the animals also contain initiator caspases that presumably activate the short pro-domain effector caspases. CARD-containing caspase-3–like proteins are rarely observed in vertebrate effector caspases. Fish-specific caspases have been found, such as the CARD-containing caspase-8 for example (28), but caspase-2 is, at present, the only characterized D*XX*Dase with a CARD.

We chose two caspases from each species to characterize further, based on the sequence comparisons with human effector caspases-3, -6, or -7. In the case of *O. faveolata*, we chose two caspase-3–like proteins that showed 47 and 35% sequence identity, respectively, with HsCasp3, and we named the two proteins OfCasp3a and OfCasp3b, respectively (Fig. 1*A* and Table 1). Interestingly, despite predicted similarity to HsCasp3, OfCasp3a also has an N-terminal CARD motif. One caspase from *P. astreoides* demonstrated the highest sequence identity with HsCasp7 (44%) and was named PaCasp7a, even though it also contains a CARD motif (Fig. 1*A* and Table 1). The second protein from *P. astreoides* showed similar sequence identity to human caspases-3, -6, -7, and -8 (36–37%) (Fig. 1*A* and Table 1), but the protein does not have a DED motif like caspase-8 and the domain organization is more similar to that of caspase-3. Consequently, we named the protein PaCasp3. Overall, the low sequence identity between the vertebrate and invertebrate caspases show that the classification is somewhat arbitrary without further biochemical characterizations of the proteins. Together, the phylogenetic analysis shows that the caspases from *P. astreoides* and *O. faveolata* have relatively low sequence identity (∼40%) to mammalian caspases as well as other vertebrate families, but the proteins had much higher sequence identities to caspases from other cnidarian species, such as *Pocillopora damicornis, Stylophora pistillata,* and *Nematostella vectensis*.

**Figure 3.**
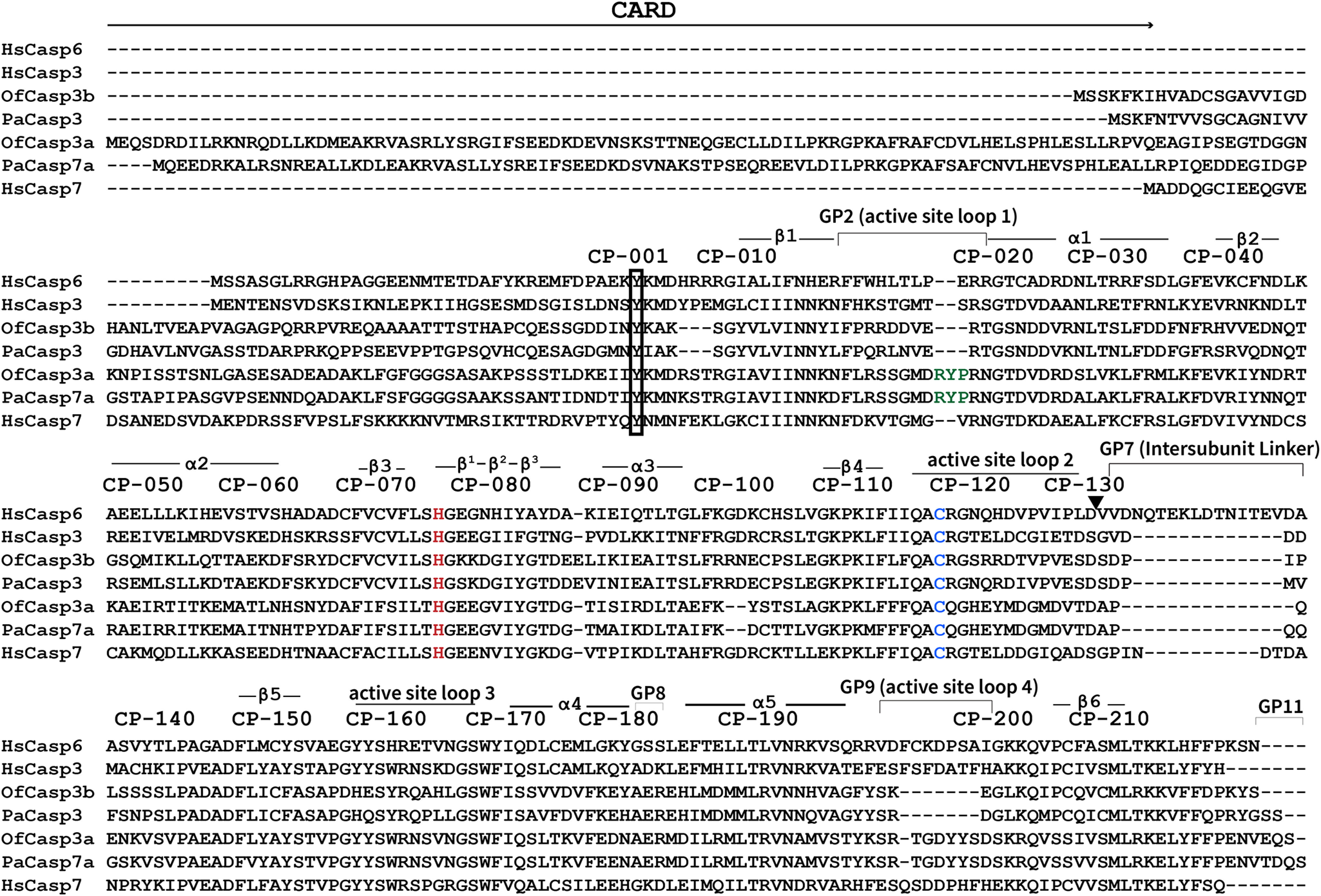
**Biochemically characterized caspases of *O. faveolata* and *P. astreoides* aligned with human effector caspases.** In the multiple sequence alignment, secondary structures (α-helices, β sheets, and loops) are indicated along with common position (*CP*) numbers among caspases. Gap positions, or sequences between common amino acid positions, are referred to as *GP*. Histidine (*H*) and cysteine (*C*), which forms a catalytic dyad, are colored in *red* and *blue* respectively. RYP motif insertions in OfCasp3a and PaCasp7a are colored in *green*.

An analysis of the coral caspase sequences shows that the proteins contain all of the conserved features that define a caspase. For example, each protein contains the catalytic dyad, histidine (CP-075) and cysteine (CP-117) (Fig. 3), where “CP” refers to the common position defined previously for caspases (29). The conserved sequence that contains the catalytic histidine (CP-115)-QACRG-(CP-119) is found in the four coral caspases, although PaCasp7a and OfCasp3a contain QACQG as in human caspase-8. One of the most highly variable regions, the intersubunit linker (IL) is the same length in OfCasp3b and PaCasp3 compared with that of HsCasp3, whereas those of PaCasp7a and OfCasp3a have one and two amino acids fewer than HsCasp3, respectively (Fig. 3).

### Biochemical characterization of coral caspases

We examined the four coral caspases by size exclusion chromatography because CARD-containing human caspases are monomers or mixtures of weak protomer-dimer (30). Because the IL of the procaspase monomer is cleaved during activation, the protomer is defined as a single unit that contains a large and small subunit and a single active site. Thus, the dimer consists of two protomers, or is more formally considered a dimer of heterodimers. The data show that the CARD containing coral caspases, PaCasp7a and OfCasp3a, elute in a single peak with molecular mass of 42.6 and 44 kDa, respectively. The sizes are larger than that of a protomer but smaller than a dimer (Fig. S2 and Table S5), suggesting that the proteins form weak dimers similar to the human initiator caspases. In contrast, the short pro-domain containing caspases, PaCasp3 and OfCasp3b, are dimers similar to the human effector caspases, with molecular mass of 64.5 and 69.2 kDa, respectively (Fig. S3 and Table S5).

We also determined the mass of the large and small subunits by MS. Caspase zymogens are cleaved in the IL, and the N-terminal CARD or pro-domain is removed during activation (30). The proteins also autoprocess during overexpression in *Escherichia coli*. The molecular size of the large and small subunits of each caspase, determined by MS, are shown in Table S5. When compared with the sequences for each protein (Fig. 3), the data show that OfCasp3a and PaCasp7a are cleaved in the intersubunit linker after (CP-127)-DVTD-(CP-130), whereas OfCasp3b and PaCasp3 are cleaved after (CP-127)-VESD-(CP-130). The actual amino acid positions, in addition to the common position number, are shown in Fig. 1*A*, and the cleavage sites are indicated by the *arrow* in Fig. 3. In addition, the first 20 or 31 amino acids, respectively, in the pro-domains of OfCasp3b and PaCasp3 are removed following cleavage after VIGD (Asp^20^) (OfCasp3b) or SSTD (Asp^31^) (PaCasp3). The CARD motifs of OfCasp3a and of PaCasp7a are removed following cleavage after DEAD (Asp^123^) and DQAD (Asp^119^), respectively (Figs. 1*A* and 3). We note that there are potentially other cleavage sites in the CARD motifs, but in our assays the CARD motif was completely removed.

**Figure 4.**
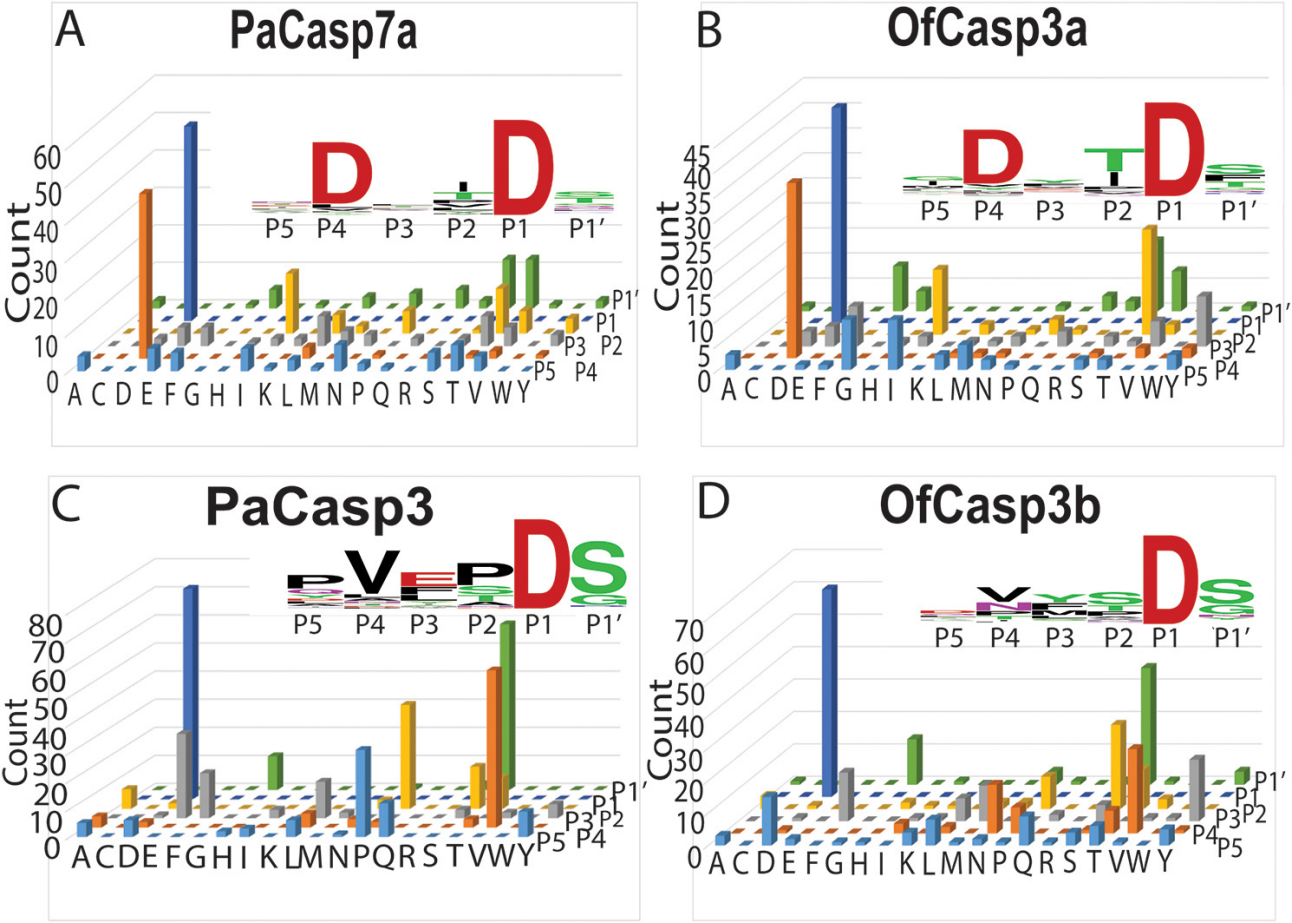
**Substrate preference determined by substrate-phage display.** *A*–*D*, amino acid preferences shown for substrate positions P5-P4-P3-P2-P1-P1′ for PaCasp7a (*A*), OfCasp3a (*B*), PaCasp3 (*C*) and OfCasp3b (*D*). Values of *y*-axes indicate number of phage sequences containing the specified amino acid (*Count*). Amino acids are shown on the *x*-axes in single letter code. Web logos are also shown in inset of respective graph for same results.

We characterized the substrate specificity for each of the four coral caspases using substrate-phage display assays, as described previously (31). In these assays, we utilize two substrate-phage libraries that determine the P5-P1′ substrate preferences, with either aspartate fixed at the P1 position (P5-*XXXX*D*X*-P1′) or random (called 6×), and the results were the same for both libraries. The data show that PaCasp7a and OfCasp3a have Group II specificity, with a preference for aspartate in the P4 position (D*XX*Dase) (Fig. 4, *A* and *B*). In contrast, PaCasp3 and OfCasp3b prefer valine in the P4 position (V*XX*Dase) (Fig. 4, *C* and D), which is defined as Group III specificity like HsCasp6.

The activities of PaCasp7a and of OfCasp3a were also examined using DEVD-AFC and VEID-AFC substrates. In all cases, however, the activity against the tetrapeptide substrates was very low because of *K_m_* values >500 μm, so we could not reliably determine the steady-state catalytic parameters *k*_cat_ or *K_m_* from the small peptide activity assays. In caspases, the *K_m_* is thought to correlate with substrate binding (*K_D_*), so the high *K_m_* suggests poor binding of the small peptide.

**Figure 5.**
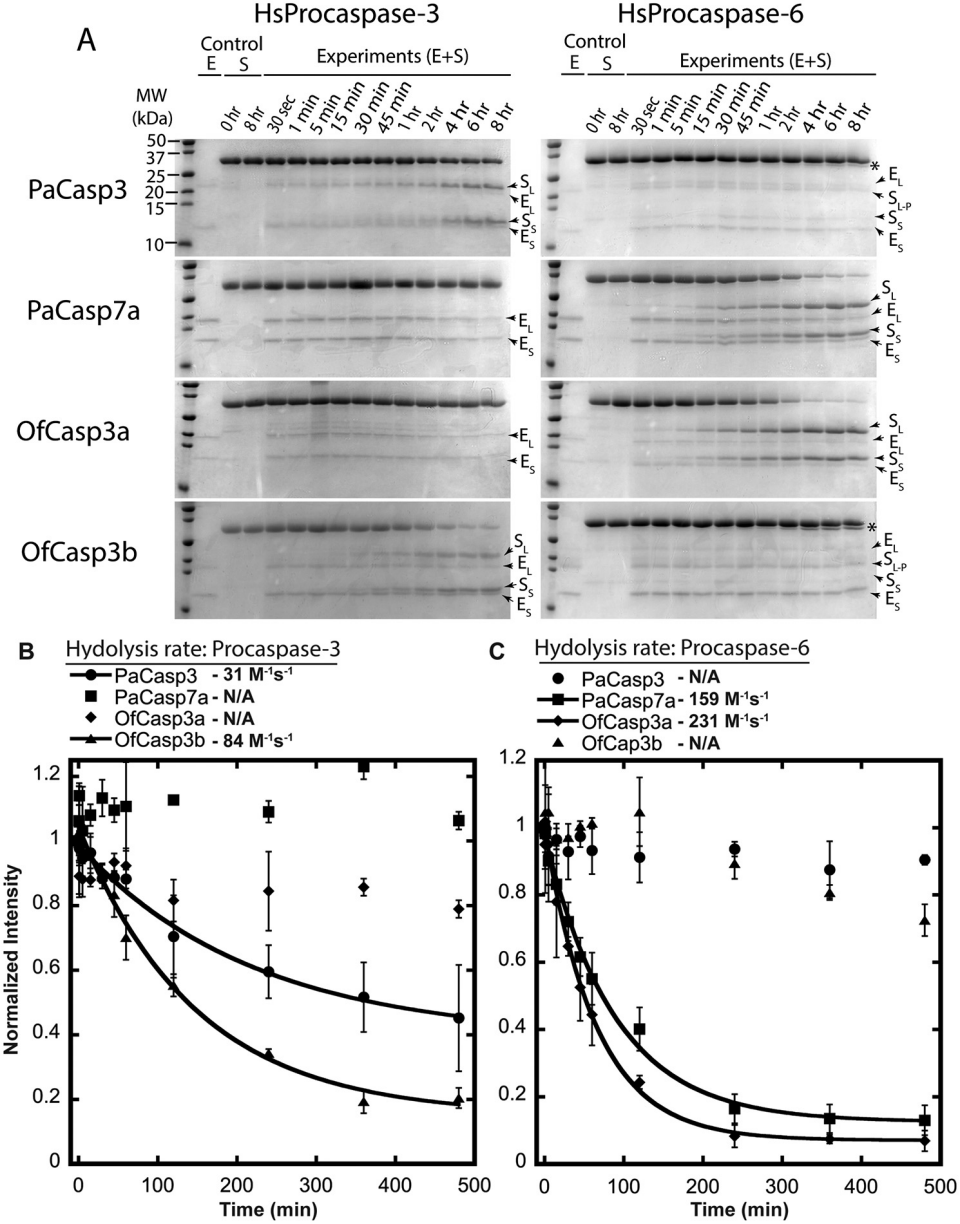
**Cleavage kinetics of coral caspases using human procaspases-3 and -6 as a substrate.** *A*, cleavage of full-length inactive HsCasp3 and HsCasp6 by coral caspases over time course. All cleaved products are labeled along with enzyme itself. (S_L_, large subunit of substrate; S_S_, small subunit of substrate; E_L_, large subunit of enzyme; E_S_, small subunit of enzyme; S_L-P_, large subunit with pro-domain cleaved; S, substrate; and E + S, enzyme and substrate). Bands with an asterisk (*) indicate only pro-domains were removed from full-length substrate. Molecular weight markers are the same for all gels. *B* and *C*, quantification of procaspase bands relative to the control (substrate without enzyme after 8 h incubation). Data were fit to a single exponential decay to calculate CF50 used to calculate hydrolysis rate of coral caspases (*solid line*). Procaspase-3 (*B*), procaspase-6 (*C*). *Error bars* represent S.D. from three different experiments.

Because of the low activity in small peptide assays, we tested the coral caspases for their ability to hydrolyze full-length human procaspases-3 and -6, which were made catalytically inactive because of mutation of the catalytic cysteine to serine (32). Thus, the proteins are incapable of undergoing self-proteolysis. As shown in Fig. 3, HsCasp3 is cleaved once in the intersubunit linker at CP-130 (IETD), whereas HsCasp6 contains two cleavage sites at CP-130 (DVVD) and at GP7-D17 (TEVD). Each procaspase substrate was incubated separately with an active coral caspase, and the reaction was monitored over 8 h. Aliquots were removed and analyzed by SDS-PAGE (Fig. 5*A*). The results show that procaspase-3 was cleaved by PaCasp3 and by OfCasp3b, with little to no cleavage by PaCasp7a or by OfCasp3a. In contrast, procaspase-6 was cleaved by PaCasp7a and by OfCasp3a, but there was little to no cleavage by PaCasp3 or by OfCasp3b. Together, the data corroborate our results from substrate-phage display (Fig. 4) that identify PaCasp3 and OfCasp3b as V*XX*Dases and PaCasp7a and OfCasp3a as D*XX*Dases, respectively.

As described previously (33), we quantified the rate of hydrolysis of the two procaspase substrates by assessing the disappearance of the full-length procaspases-3 and -6, both ∼32 kDa in size, and the appearance of the large (∼20 kDa) and small (∼10 kDa) subunits over the time course of the assay (Fig. 5, *B* and *C*). The data were fit to a single exponential decay to approximate *k*_cat_/*K_m_*. The results show that PaCasp3 and OfCasp3b cleave procaspase-3 with hydrolysis rates of 31 m^−1^s^−1^ and 84 m^−1^s^−1^, respectively (Fig. 5*B*), whereas PaCasp7a and OfCasp3a cleaved procaspase-6 with hydrolysis rates of 159 M^−1^s^−1^ and 231 M^−1^s^−1^, respectively (Fig. 5*C*). We note that, although not quantified, both PaCasp3 and OfCasp3b cleave the procaspase-6 pro-peptide (TETD) at a much slower rate than that observed for cleavage of the intersubunit linker of procaspase-3 (IETD). Together, the biochemical data show that the coral caspases are weak enzymes, at least in the *in vitro* assays, with *k*_cat_/*K_m_* values ∼10^2^ m^−1^s^−1^.

### Crystal structure of PaCasp7a

**Figure 6.**
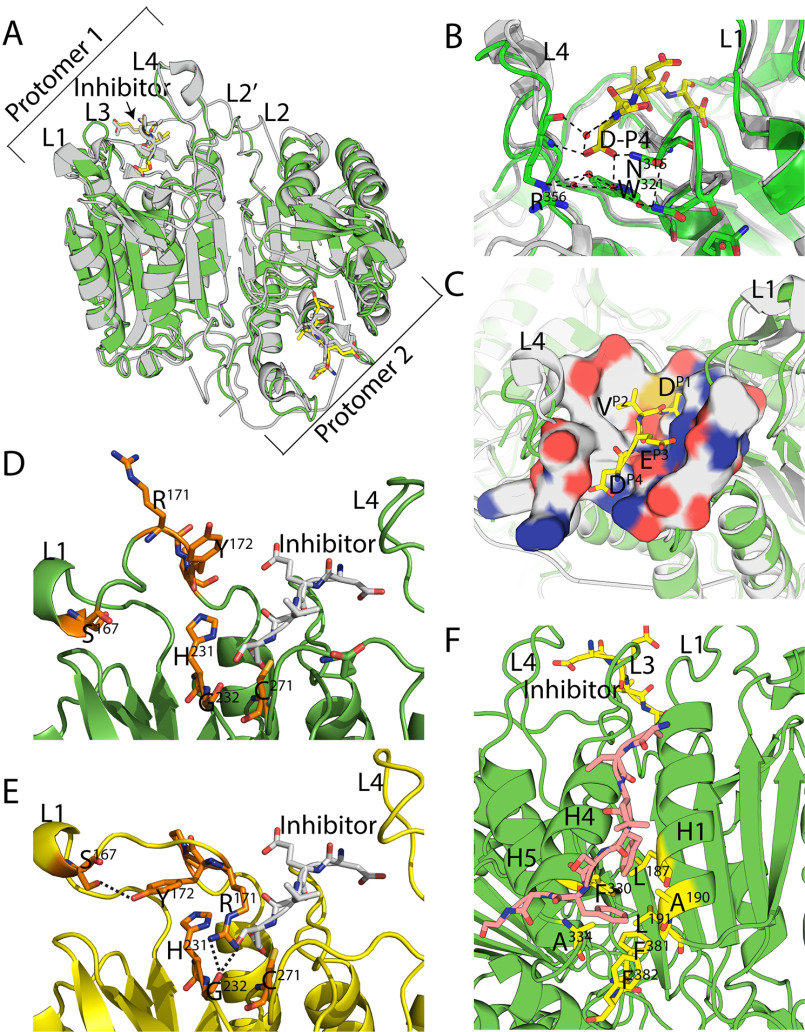
**Structure of PaCasp7a.** *A*, comparison of PaCasp7a (*green*) aligned with HsCasp3 (*gray*) (PDB ID: 2J30). *B*, PaCasp7a active site bound with inhibitor DEVD-CHO. *Dashed lines* show hydrogen bonding network to the P4 aspartate. *C*, surface map of active site residues in PaCasp7 within 5 Å of the inhibitor (*yellow sticks*). Neutral charges are *gray*, negative charges are *red*, and positive charges are *blue*. *D*, “out” orientation, of RYP residues in loop 1 in crystal structure of PaCasp7a. *E*, “in” orientation, of RYP residues in loop 1 in predicted model of PaCasp7a. *Dashed lines* show the hydrogen bonds formed by R and Y in In orientation. *F*, N-terminal peptide (*orange*) bound in hydrophobic pocket between helices 1 and 4. PaCasp7a residues that form the pocket are shown in *yellow*: Leu^187^ (CP-031), Ala^190^ (CP-034), Leu^191^ (CP-035), Phe^330^ (CP-177), Ala^334^ (CP-181), as well as Phe^381^ (CP-217) and Phe^382^ (CP-218) at the C terminus.

We attempted to crystalize all four of the coral caspases, and we were successful in obtaining diffraction quality crystals of PaCasp7a with an inhibitor (DEVD-CHO) bound in the active site. The crystals diffracted in the P2_1_2_1_2_1_ space group, and we determined the structure to high resolution at 1.57 Å (Table S4). The data show that the PaCasp7a is very similar to human caspases, with a root mean square deviation of <1 Å compared with HsCasp3 (Fig. 6*A*). In the active site, the carboxylate group of the P4 aspartate hydrogen bonds to Asn^315^ (CP-162) on active site loop 3 (L3), the backbone amide of Arg^356^ (GP9-02) on L4, and through water–hydrogen bonds to Trp^321^ (CP-168) (on L3) as well as the backbone carbonyl of Arg^356^ (GP9-02) (on L4) (Fig. 6*B*). In general, the active site provides hydrophilic binding pockets for the P3 glutamate and P4 aspartate of the substrate, and a more hydrophobic binding pocket for the P2 valine side chain (Fig. 6*C*), similarly to that of HsCasp3.

Both PaCasp7a and OfCasp3a contain a two-residue insertion in loop 1 (L1) of the active site (Fig. 3). The structure of PaCasp7a with inhibitor bound shows that the insertion extends the loop compared with HsCasp3 and results in an “RYP” motif in L1 (Fig. 3) near the catalytic histidine (Fig. 6*D*). Models of the PaCasp7a active site suggest that rotation of the loop results in intercalation of the Arg^171^ (GP2-17) between the catalytic His^231^ (CP-075) and Cys^271^ (CP-117) (Fig. 6*E*). In this orientation, the arginine side-chain hydrogen bonds with the carbonyl of Gly^232^ (CP-076) on active site loop 3 (L3) and clashes with the P1 and P2 positions of substrate. In addition, the tyrosine of the RYP motif forms a new hydrogen bond with the side chain of Ser^167^ (GP2-05) in L1 (Fig. 6*E*). Altogether, the models suggest that in the absence of substrate, rotation in L1 may stabilize an inactive conformation of the enzyme. We note, however, that MD simulations (50 ns) of the structural models show that the region of L1 that contains the RYP motif is very mobile, so if the RYP motif is indeed autoinhibitory, then the “RYP-In” conformation appears to be transient (Fig. S3).

The structure of PaCasp7a also reveals a peptide bound on the protein surface near α-helices 1 and 4. The structure shows that amino acids in the N terminus of PaCasp7a (N′-AKLFSFGG-C′) (N′-PD-A025 to PD-G018-C′ in the common position numbering) compose the peptide, where the two phenylalanine side chains bind in a hydrophobic pocket between helices 1 and 4 (Fig. 6*F*). The binding pocket on the protein is formed by five hydrophobic residues on the two helices (Leu^187^ (CP-031), Ala^190^ (CP-034), Leu^191^ (CP-035), Phe^330^ (CP-177), Ala^334^ (CP-181)) as well as Phe^381^ and Phe^382^ (CP-217 and CP-218) at the C terminus (Fig. 6*F*). The peptide also forms several hydrogen bonds with charged groups on the protein surface. We do not observe electron density for amino acids Gly^128^ (PD-017) to Asn^141^ (PD-004) (Fig. 3), but extensive interactions downstream of Asp^142^ (PD-003) result in an ordered structure that moves into the core of the protein. The 14 disordered residues would provide ample distance to connect the peptide with the protease domain, and the data suggest that the intervening amino acids may hinder dimerization because they would be anchored near the dimer interface when the peptide is bound on the protein surface. The N-terminal end of the peptide is immediately downstream of the DQAD cleavage site that removes the CARD motif (Fig. 3), suggesting that the binding pocket on the protein surface may be used to position the N-terminal linker (between the CARD and protease domains) in the active site.

We searched the caspase structures in the Protein Data Bank and found four examples of an N-terminal peptide bound between helices 1 and 4: human caspases-1 (PDB ID 2H48) and -2 (PDB ID 3R7M), Dronc of *Drosophila melanogaster* (PDB ID 2FP3), and CED-3 of *Caenorhabditis elegans* (PDB ID 4M9R) (34–37) (Fig. S4, *A–D*). Interestingly, the region of the peptide that is disordered in PaCasp7a (Gly^128^ PD-017 to Asn^141^ PD-004) forms a short α-helix in caspases-1 and -2 and in CED-3 (Fig. S4, *A*, *B*, and *D*). The short helix does not make contacts across the dimer interface but rather makes extensive intraprotomer contacts with the C terminus of the protein. In the case of DRONC, the intervening peptide forms an extended structure that extends beyond the dimer interface and would clash with the second protomer of the dimer (Fig. S4*C*). In all cases, the binding pocket between helices 1 and 4 is hydrophobic, and the peptide binds through insertion of one or more hydrophobic amino acids into the binding pocket as well as hydrogen bonds between side chains on the protein surface and backbone atoms of the peptide. Therefore, the structures show a common theme in which the N-terminal peptide downstream of the pro-domain cleavage site binds to a hydrophobic pocket on the protein surface. The interactions likely stabilize the peptide in the binding pocket for cleavage.

Finally, we observed a similar hydrophobic pocket in human effector caspases (HsCasp3, PDB ID: 2J30; HsCasp6, PDB ID: 3S70; HsCasp7, PDB ID: 1F1J) (38–40) (Fig. S4, *E–G*). There is no evidence, however, from biochemical or structural data that the N-terminal peptide of the short pro-domain caspases bind in the hydrophobic pocket. A comparison of the N-terminal sequences (Fig. 3) shows significant divergence in the peptide of human effector caspases, so although the binding pocket is similar to that of PaCasp7a, the binding interactions with the peptide sequences are not similar. In HsCasp3 and HsCasp6, for example, the cleavage site is downstream of the putative binding sequence, so the entire peptide is removed from the N terminus. Interestingly, in HsCasp7 the cleavage site is upstream of the binding region, but the sequence evolved into a tetra-lysine motif that has been shown to be an exosite for substrate selection in caspase-7 (41).

## Discussion

Coral reefs are facing a significant decline because of increasing local and global stressors from disease, climate change, and pollution (42). Although coral possess a robust innate immune system, the cellular mechanisms these organisms use to respond to disease have not been functionally characterized (43). In addition, elevated ocean temperatures have emerged as key threats to the long-term survival of coral reefs because of collapse of the coral–algal symbiosis (23, 42). Given the environmental consequences posed by coral disease and bleaching, understanding the molecular physiology behind coral immune responses will improve our understanding of coral declines (44). The two coral species used in this study *Orbicella faveolata* and *Porites astreoides* are both reef-building coral, but they lie on opposite ends of the disease-response spectrum. Where *O. faveolata* is sensitive to disease and activates apoptotic responses to stress, *P. astreoides* is resistant to disease and activates autophagic responses to stress. Thus, these two species of coral represent an intriguing system to understand and characterize apoptotic mechanisms (21).

The phylogenetic analysis suggests that the caspase repertoire of reef-building corals cluster into two categories: coral effectors and coral initiators (Fig. 2). This indicates that in contrast to model systems such as *C. elegans* and *Drosophila,* corals likely possess caspases with discrete functions that mirror vertebrate caspases. Protein domain predictions for the caspase repertoire from our two focal species determined that the sequences in the initiator caspase family possess N-terminal CARD or DED domains similar to human caspases, whereas the predicted coral effector caspases contain short pro-domains, also a pattern reflected in human caspases. Overall, this comparative analysis demonstrates that it is likely that corals possess complex apoptosis networks similar to vertebrates.

Our functional characterization suggests that PaCasp7a and OfCasp3a may function similarly to caspase-2 because they exhibit D*XX*Dase activity and contain an N-terminal CARD motif. In contrast, PaCasp3 and OfCasp3b share characteristics with effector caspase-6, with a short pro-domain and V*XX*Dase activity. Moreover, a phylogenetic analysis showed that OfCasp3a and PaCasp7a are close to vertebrate initiator caspases, whereas PaCasp3 and OfCasp3b are closer to effector caspases. Although the caspases exhibited low activity against peptide substrates, we were able to confirm the selection through cleavage assays of protein substrates. The results showed that the D*XX*Dases (PaCasp7a and OfCasp3a) processed procaspase-6, which has a DVVD cleavage sequence in the intersubunit linker, but not procaspase-3, which contains a more hydrophobic recognition sequence recognized by caspase-8 (IETD). The opposite was true for PaCasp3 and OfCasp3b. In those cases, the enzymes processed procaspase-3 but not procaspase-6.

**Figure 7.**
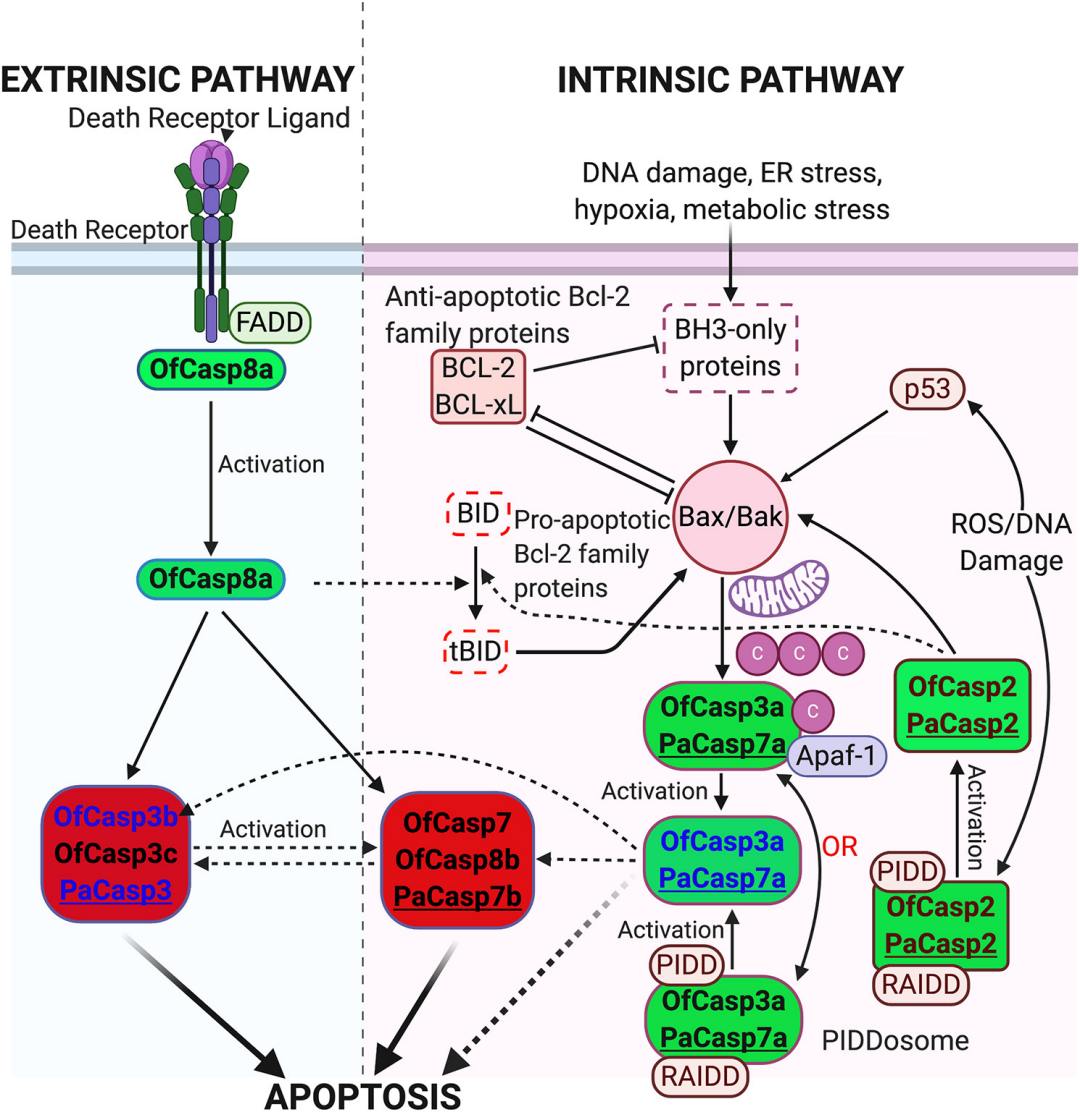
**Proposed apoptotic pathways in coral compared with the apoptotic pathways in humans.** All components in the pathways have homologs in *O. faveolata* and *P. astreoides* with the exception of BID (*dotted box*). A list of homologs is shown in Table S6. *Dotted lines* indicate that links have not yet been shown experimentally. Caspases in *green* background are initiators and those in *red* background are effectors. *Pa* refers to *P. astreoides* and *Of* refers to *O. faveolata.* The four caspases characterized here are shown in *blue*.

The biochemical data show that the two short prodomain caspases (OfCasp3b and PaCasp3) are most likely not the main executioner caspases in coral. Rather, the enzymes may function similarly to HsCasp6, which plays a role during cell development. In addition, the two effector caspases are not directly activated by the CARD-containing caspases, PaCasp7a or OfCasp3a. At present, it is not clear if the D*XX*Dase activity of the two effector caspases functions as the primary executioner of apoptosis or if the proteins activate the as-yet-unidentified executioner caspase. Based on the caspases identified in coral, we suggest a model in which the PaCasp7a and OfCasp3a enzymes are activated on PIDDosome-like complexes (45, 46), similar to HsCasp2 (Fig. 7). Either the D*XX*Dase activity is utilized to kill cells, like CED-3 in *C. elegans*, or the activated enzymes cleave the executioner. In the latter case, the PaCasp3 and OfCasp3b proteins would be indirectly activated by PaCasp7a and OfCasp3a, respectively, through the undefined executioner caspase. Alternatively, the OfCasp3a and PaCasp7a may be activated on apoptosome complexes, and the D*XX*Dase activities could be used to activate downstream caspases or to execute apoptosis. The latter suggestion is consistent with the presence of coral caspase-2–like proteins that also contain CARD motifs (Fig. 1*A*). Caspase-8–like proteins containing DED motifs would also activate the executioner (and PaCasp3 or OfCasp3b) indirectly. The putative caspase-8 and caspase-3 executioner proteins have been identified, but not yet characterized, in coral (47–50). Further characterization of the apoptotic components will determine the signaling responses leading to activation of the caspases, in particular whether OfCasp3a and PaCasp7a are activated on PIDDosome or apoptosome complexes. Together, the data suggest that coral are responsive to death ligands as well as metabolic changes in the cell, matching human extrinsic and intrinsic apoptotic pathways and establishing their importance in the context of coral health, disease, and bleaching.

Finally, the data shown here for PaCasp7a, as well as previous structural data, human caspases-1 (PDB ID 2H48) and -2 (PDB ID 3R7M), Dronc of *Drosophila melanogaster* (PDB ID 2FP3) and CED-3 of *Caenorhabditis elegans* (PDB ID 4M9R), and human effector caspases (HsCasp3, PDB ID: 2J30; HsCasp6, PDB ID: 3S70; HsCasp7, PDB ID: 1F1J) (34–40) (Fig. S4, *A–G*), identify a hydrophobic pocket on the protein surface between helices 1 and 4 in which a peptide sequence C-terminal to the processing sequence binds. The binding of the peptide may increase activity through improved binding of the recognition sequence in the active site and help position the linker near the CARD-motif for cleavage. Alternatively, the binding of the peptide to the pocket may affect substrate selection by demonstrating a preference for substrates with both a P1-P4 cleavage sequence and the downstream sequence that binds to the pocket. We also showed that the hydrophobic pocket is conserved in a wide range of species, with similar size and properties. The short pro-domain caspases appear to have retained the binding pocket on the protease domain, but the N-terminal peptide sequence diverged, suggesting that effector caspases may utilize the binding pocket as an exosite for substrate selection. In this case, for example, substrates with sequences that bind in the pocket, and are downstream of the cleavage site, may exhibit better binding compared with substrates that contain only the P1-P4 recognition sequences.

## Conclusions

Coral have complex apoptotic signaling cascades, similar to those of vertebrates, providing another line of evidence that apoptotic mechanisms are ancient and well conserved in metazoans. We have identified OfCasp3a and PaCasp7a as initiator caspases that appear to function similarly to HsCasp2, indicating that corals are responsive to metabolic changes in the cell. In addition, both *O. faveolata* and *P. astreoides* contain V*XX*Dases similar to HsCasp6. Our data show that the enzymes from both species have similar biochemical properties and are activated by similar mechanisms. Given that coral caspases have relatively low sequence identity to human caspases, the designation of caspase function based on sequence similarity should undergo further biochemical characterization. Together, the data show that regulatory or physiological mechanisms rather than differences in the caspase cascade likely dictate the disease sensitivity of *O. faveolata* or disease resistance of *P. astreoides.* Potential differences could arise from response mechanisms in the death receptor or the PIDDosome activation platforms upstream of the caspase cascade. Our data suggest that differences in the receptor-mediated activation of caspases as well as crosstalk between the autophagic and apoptotic pathways in the two coral species lead to the different physiological responses.

## Experimental Procedures

### Cloning, protein expression, and protein purification

The codon optimized sequences of the four coral caspases PaCasp3, PaCasp7a, OfCasp3a, and OfCasp3b were based on the sequences from previous transcriptomic data (19) and were cloned into pET11a vector (GenScript, Piscataway, NJ, USA). All proteins contained a C-terminal His_6_ tag and were expressed in *E. coli* BL21(DE3) pLysS cells and purified as described previously (32, 51).

### Phylogenetic analysis

Caspase sequences of representative species were obtained from the CaspBase (RRID:SCR_018975) (29) along with BLAST top hits from HMMER (52), and multiple sequence alignments were obtained using MEGA 7 (53). The best model of evolution to construct a phylogenetic tree from our dataset was determined with ProtTest 3 (54) (https://github.com/ddarriba/prottest3), and the tree was computed with the maximum likelihood method in IQTREE, using the Jones-Taylor Thornton model plus γ distribution (55). The tree was bootstrapped 1000 times as a test of phylogeny. The accession numbers of all genes used for phylogenetic analysis are listed in Tables S1 and S2.

### Size exclusion chromatography

Proteins were examined using a Superdex75 Increase 10/300GL column on an AKTA-FPLC. The proteins were concentrated to 1–5 mg/ml and dialyzed in a buffer of 30 mm potassium phosphate, pH 7.5, containing 1 mm DTT for 4 h. The column was equilibrated with 2 column volumes (50 ml) of the same buffer. Protein (200 μl) was loaded onto the column, and the column was resolved at a flow rate of 0.5 ml/min. The column was calibrated using the gel filtration LMW calibration kit (GE Health Sciences) following the manufacturer instructions.

### MS

MALDI analysis was done as described (56). In brief, proteins were resolved by SDS-PAGE on a 12.5% acrylamide gel, and then bands for the large and small subunits were excised. Each gel fragment was destained using a solution of acetonitrile and 50 mm ammonium bicarbonate (1:1 v/v) for 3 h. The gel fragments were then crushed in microcentrifuge tubes, and the proteins were extracted with 30 μl of a solution of formic acid/water/2-propanol (1:3:2 v/v/v) for 8 h at room temperature. After extraction, samples were centrifuged and supernatant was lyophilized then re-dissolved in 2 μl of MALDI matrix solution (formic acid/water/2-propanol saturated with 4-hydroxy-α-cyano-cinnamic acid). Dissolved protein was then retrieved for MS analysis using dried-drop method of matrix crystallization then analyzed by MALDI-MS (Axima Assurance Linear MALDI TOF).

### Whole-protein cleavage assay

Enzyme specificity of the four coral caspases was first examined by cleavage of human procaspases-3 and -6 in time-course assays, as described previously (33). The procaspase substrate was diluted to a final concentration of 5 μm in a buffer of 150 mm Tris-HCl, pH 7.5, 50 mm NaCl, 1% sucrose, and 10 mm DTT at 37°C. Reactions were started by the addition of respective coral caspase at a final concentration of 1 μm, and the total reaction volume was 2 ml. Aliquots of 100 μl were removed at times 30 s, 1 min, 5 min, 15 min, 30 min, 45 min, 1 h, 2 h, 4 h, 6 h, and 8 h after the addition of active enzyme. Reactions were stopped by the addition of 6-fold concentrated SDS-PAGE loading dye (20 μl) followed by incubation in boiling water for 5 min. Samples were loaded into a 16% resolving gel with a 4% stacking gel and electrophoresed for 1.5 h at 80 volts followed by an increase in voltage to 190 V for an additional 4 h. The change in density for the procaspase substrate over time as a result of cleavage was quantified using Image laboratory software (Bio-Rad), and the data were plotted with Kaleidagraph. As described previously (33), the data were fit to an exponential decay to determine the CF50 (cleavage of 50% of protein substrate), and the CF50 was used to calculate the rate of hydrolysis (m^−1^ s^−1^) using the equation *k* = ((−ln(*P*))/(*E*·*t*)). In this case, *k* is the rate of hydrolysis, *P* is the fraction cleaved (50%), *E* is the concentration at which CF50 is achieved (in molar), and *t* represents time (in seconds).

### Enzyme assays and substrate-phage display

Enzyme activity was determined in a buffer of 150 mm Tris-HCl, pH 7.5, 50 mm NaCl, 10 mm DTT, 1% sucrose, 0.1% CHAPS (assay buffer) at 25°C, as described previously (57, 58). The total reaction volume was 200 μl, and the final enzyme concentration was 10 nm. Following the addition of substrate (Ac-DEVD-AFC, Ac-VEID-AFC, Ac-LETD-AFC, Ac-LEHD-AMC, Ac-IETD-AMC), the samples were excited at 400 nm (AFC substrates) or 350 nm (AMC substrates), and fluorescence emission was monitored at 505 nm (for AFC substrates) or 450 nm (for AMC substrates) for 60 s using a PTI fluorometer (Photon Technology International, Edison, NJ, USA). The steady-state parameters, *K_m_* and *k*_cat_, were determined from plots of initial velocity *versus* substrate concentration.

Substrate phage display assays were performed as described (31, 58). Briefly, phage libraries consisting of caspase recognition sequences were bound to Ni-NTA resin. Enzyme (10–100 nm) was added to initiate the reaction, and samples were incubated between 3 and 20 h. *E. coli* ER2738 cells were used to amplify the cleaved phage from previous rounds by infecting cells with the supernatant after enzyme incubation. The cells were grown for 4 h and removed by centrifugation, and the supernatant was collected and used as the library for the following round of selection. Plaque counting was used to determine the end point of the experiment, when the number of phage bound to the resin was similar to the number of phage released during the treatment. The number of phage released during the reaction *versus* the control (without enzyme) was monitored to ensure progress in substrate selectivity.

### X-ray crystallography

Protein structure predictions were performed using Swiss-Model (59) using human caspases-3, -6, and -7 as references (PDB IDs 2J30, 3OD5, and 1F1J, respectively). For structure determination, the coral caspase proteins were dialyzed in a buffer of 10 mm Tris-HCl, pH 7.9, 100 mm NaCl, 1 mm DTT and concentrated to ∼7 mg/ml. The molar extinction coefficients for the proteins were determined by ProtParam under reduced conditions (Table S3). Inhibitor, Ac-DEVD-CHO (reconstituted in DMSO), was added at a 5:1 (w/w) inhibitor/protein ratio, and DTT and NaN_3_ were added to final concentrations of 10 and 3 mm, respectively. Samples were incubated for 1 h in the dark on ice. Hanging-drop vapor diffusion method was applied using 4 μl drops that contained equal volumes of protein and reservoir solutions using the PEG/ion 2 screen (Hampton Research). PaCasp7a protein crystalized in a solution of 0.1 m sodium malonate, pH 5.0, 12% w/v PEG 3350, and conditions were optimized such that the best diffracting crystals of PaCasp7a were obtained at 18°C in a solution of 0.1 m sodium malonate, pH 4.9–5.1, 15–17% PEG 3350 (w/v), 10 mm DTT, and 3 mm NaN_3_. Crystals for PaCasp7a appeared within 3 to 5 days and were briefly immersed in a cryogenic solution containing 20% PEG 4000, 80% reservoir solution. Crystals were stored in liquid nitrogen. We were unable to obtain diffraction quality crystals for the remaining coral caspases. Datasets were collected at 100 K at the SER-CAT synchrotron beamline (Advance Photon Source, Argonne National Laboratory, Argonne, IL, USA). Each dataset contained 180 frames at 1-degree rotation. The protein crystallized in the space group P 2_1_ 2_1_ 2_1_ and was phased with a previously published HsCasp3 structure (PDB ID 2J30). Data reduction and model refinements were done using HKL2000, COOT, and Phenix, and a summary of the data collection and refinement statistics is shown in Table S4. Molecular dynamics simulations were performed for 50 ns with GROMACS 4.5 (60) using the Amber99 force field (61) and the TIP3P water model (62), as described previously (63).

## Data availability

The crystal structure for PaCasp7a has been deposited in the Protein Data Bank under PDB ID: 6WI4.

## Supplementary Material

Supporting Information
