# Supplementary material for: Caspases from scleractinian coral show unique regulatory features
Source: J Biol Chem. 2020 Aug 11;295(43):14578–91. doi: 10.1074/jbc.RA120.014345 (PMC7586219; doi:10.1074/jbc.RA120.014345)
Supplement: Supporting Information [file supp_295_43_14578__index.html]

Caspases from scleractinian coral show unique regulatory features — Characterization of coral caspases — Supporting Information 

# Caspases from scleractinian coral show unique regulatory features

## Supporting Information

- Supporting Information (to be published online) - Supplemental figures and tables2.pdf
